# Supplementary material for: Time-series transcriptome analysis identified differentially expressed genes in broiler chicken infected with mixed Eimeria species
Source: Front Genet. 2022 Aug 8;13:886781. doi: 10.3389/fgene.2022.886781 (PMC9393255; doi:10.3389/fgene.2022.886781)
Supplement: Supplementary file 2 [file DataSheet1.ZIP › 4dpi_KEGG.Gsea.1625100298584/KEGG_HOMOLOGOUS_RECOMBINATION.html]

Details for gene set KEGG\_HOMOLOGOUS\_RECOMBINATION[GSEA]

|  || Dataset | TMM\_4dpi\_gct\_format\_4dpi\_gct\_format.Class\_4dpi.cls #PC\_versus\_NC.Class\_4dpi.cls #PC\_versus\_NC\_repos |
| Phenotype | Class\_4dpi.cls#PC\_versus\_NC\_repos |
| Upregulated in class | 1 |
| GeneSet | KEGG\_HOMOLOGOUS\_RECOMBINATION |
| Enrichment Score (ES) | 0.5252371 |
| Normalized Enrichment Score (NES) | 1.5232716 |
| Nominal p-value | 0.04364798 |
| FDR q-value | 0.27575964 |
| FWER p-Value | 0.951 |
Table: GSEA Results Summary

  

Fig 1: Enrichment plot: KEGG\_HOMOLOGOUS\_RECOMBINATION      
 Profile of the Running ES Score & Positions of GeneSet Members on the Rank Ordered List

  

| SYMBOL | TITLE | RANK IN GENE LIST | RANK METRIC SCORE | RUNNING ES | CORE ENRICHMENT || 1 | RAD52 | na | 490 | 0.973 | 0.0672 | Yes |
| 2 | RPA3 | na | 637 | 0.856 | 0.1501 | Yes |
| 3 | NBN | na | 735 | 0.796 | 0.2305 | Yes |
| 4 | RAD54B | na | 768 | 0.775 | 0.3139 | Yes |
| 5 | RAD50 | na | 816 | 0.753 | 0.3936 | Yes |
| 6 | EME1 | na | 922 | 0.701 | 0.4627 | Yes |
| 7 | RAD51 | na | 1046 | 0.655 | 0.5252 | Yes |
| 8 | RPA2 | na | 3366 | 0.236 | 0.3580 | No |
| 9 | MRE11 | na | 3566 | 0.211 | 0.3648 | No |
| 10 | RAD51C | na | 3935 | 0.174 | 0.3534 | No |
| 11 | RAD51D | na | 4377 | 0.136 | 0.3317 | No |
| 12 | RPA1 | na | 4548 | 0.120 | 0.3309 | No |
| 13 | BRCA2 | na | 5486 | 0.038 | 0.2569 | No |
| 14 | TOP3A | na | 5502 | 0.037 | 0.2598 | No |
| 15 | POLD2 | na | 5963 | -0.003 | 0.2218 | No |
| 16 | TOP3B | na | 6143 | -0.018 | 0.2088 | No |
| 17 | RAD54L | na | 6277 | -0.029 | 0.2009 | No |
| 18 | BLM | na | 6769 | -0.068 | 0.1675 | No |
| 19 | XRCC3 | na | 8719 | -0.253 | 0.0330 | No |
| 20 | SSBP1 | na | 9368 | -0.331 | 0.0157 | No |
| 21 | XRCC2 | na | 9637 | -0.367 | 0.0341 | No |
| 22 | POLD3 | na | 11081 | -0.631 | -0.0162 | No |
| 23 | RAD51B | na | 11555 | -0.843 | 0.0380 | No |
Table: GSEA details [plain text format]

  

Fig 2: KEGG\_HOMOLOGOUS\_RECOMBINATION      
 Blue-Pink O' Gram in the Space of the Analyzed GeneSet

  

Fig 3: KEGG\_HOMOLOGOUS\_RECOMBINATION: Random ES distribution      
 Gene set null distribution of ES for **KEGG\_HOMOLOGOUS\_RECOMBINATION**
